# Supplementary material for: Epigenetic models developed for plains zebras predict age in domestic horses and endangered equids
Source: Commun Biol. 2021 Dec 17;4:1412. doi: 10.1038/s42003-021-02935-z (PMC8683477; doi:10.1038/s42003-021-02935-z)
Supplement: Supplementary file 2 — Supplementary Information [file 42003_2021_2935_MOESM2_ESM.pdf]

Supplemental Information for:

Epigenetic models developed for plains zebras predict age in domestic horses and endangered equids

Brenda Larison[1,2a], Gabriela M. Pinho[1a], Joseph A. Zoller[3], Amin Hagani[3], Caesar Z. Li[3], Carrie Finno[4], Colin Farrell[5], Matteo Pellegrini[5], Christopher B. Kaelin[6,7], Gregory S. Barsh[6,7], Bernard Wooding[8], Steve Horvath[3,9]

[1]Department of Ecology and Evolutionary Biology, UCLA, Los Angeles, CA, 90095, USA
[2]Center for Tropical Research, Institute for the Environment and Sustainability, UCLA, Los Angeles CA, 90095, USA
[3]Human Genetics, David Geffen School of Medicine, University of California, Los Angeles CA 90095, USA
[4]School of Veterinary Medicine, University of California, Davis CA 95616, USA
[5]Department of Molecular, Cell and Developmental Biology, University of California, Los Angeles CA, USA
[6]HudsonAlpha Institute for Biotechnology, Huntsville AL, 35806, USA
[7]Department of Genetics, Stanford University, Stanford CA, 94305, USA
[8]Quagga Project, Elandsberg Farms, Hermon, 7308, South Africa
[9]Department of Biostatistics, Fielding School of Public Health, University of California, Los Angeles, Los Angeles CA, USA
[a]Both authors contributed equally

Supplementary Table 1: Results from the linear models to test whether inbreeding is associated with epigenetic aging. Results from the full data set (RAD + imputed, n = 69) are shown in panels **a-d**. Results from RADseq only (n=42) are shown in panels **e-h**. Age acceleration was the dependent variable and was calculated from Epigenetic Pacemaker models (**a**, **c** and **e**, **g**) and from Epigenetic clock models (**b**, **d** and **f**, **h**). Inbreeding was estimated as F (**a**, **b**, and **e**, **f**) and F_{ROH} (**c**, **d** and **g**, **h**) in PLINK. Individual inbreeding level, sex and chronological age as well as the interaction between the inbreeding measure and chronological age were fixed effects.

RAD + Imputed

EPM age acceleration

Inbreeding measure: F

a

	Estimate ± SE	t	P	
Intercept	-0.578 ± 0.601	-0.962	0.3396	
F	-1.007 ± 2.734	-0.369	0.7137	
Chron. Age	0.103 ± 0.105	0.981	0.3302	
Sex	0.219 ± 0.432	0.507	0.6140	
F:Chron. Age	1.281 ± 0.541	2.366	0.0211	*

Inbreeding measure: F_{ROH}

c

	Estimate ± SE	t	P	
Intercept	0.707 ± 0.706	1.001	0.3204	
F_{ROH}	-9.772 ± 3.252	-3.005	0.0038	**
Chron. Age	-0.159 ± 0.073	-2.186	0.0325	*
Sex	0.162 ± 0.459	0.353	0.7249	
F_{ROH}:Chron. Age	2.670 ± 0.886	3.011	0.0037	**

EC age acceleration

Inbreeding measure: F

b

	Estimate ± SE	t	P	
Intercept	0.271 ± 0.1034	2.624	0.0109	*
F	0.499 ± 0.551	0.906	0.3686	
Chron. Age	-0.125 ± 0.049	-2.552	0.0131	*
Sex	-0.064 ± 0.058	-1.115	0.2692	
F:Chron. Age	-0.110 ± 0.367	-0.301	0.7641	

Inbreeding measure: F_{ROH}

d

	Estimate ± SE	t	P	
Intercept	0.306 ± 0.115	2.654	0.01	*
F_{ROH}	0.116 ± 0.708	0.164	0.8705	
Chron. Age	-0.133 ± 0.056	-2.381	0.0203	*
Sex	-0.069 ± 0.059	-1.170	0.2464	
F_{ROH}:Chron. Age	-0.080 ± 0.501	-0.159	0.8743	

RADseq only

EPM age acceleration

Inbreeding measure: F

e

	Estimate ± SE	t	P	
Intercept	-1.518 ± 0.594	-2.553	0.0149	*
F	-1.696± 3.911	-0.434	0.6672	
Chron. Age	0.270 ± 0.063	4.312	0.0001	***
Sex	0.118 ± 0.518	0.229	0.8205	
F:Chron. Age	2.321 ± 0.416	5.573	2.4E-06	***

Inbreeding measure: F_{ROH}

g

	Estimate ± SE	t	P	
Intercept	0.813 ± 0.768	1.058	0.2971	
F_{ROH}	-21.634 ± 5.542	-3.904	0.0004	***
Chron. Age	-0.117 ± 0.071	-1.644	0.1086	
Sex	-0.386 ± 0.562	-0.688	0.4960	
F_{ROH}:Chron. Age	5.690 ± 1.294	4.397	8.9E-05	***

EC age acceleration

Inbreeding measure: F

f

	Estimate ± SE	t	P	
Intercept	0.154 ± 0.104	1.483	0.1466	
F	0.909 ± 0.800	1.135	0.2635	
Chron. Age	-0.067 ± 0.039	-1.718	0.0941	.
Sex	-0.142 ± 0.078	-1.820	0.0769	.
F:Chron. Age	-0.223 ± 0.423	-0.527	0.6017	

Inbreeding measure: F_{ROH}

h

	Estimate ± SE	t	P	
Intercept	0.282 ± 0.143	1.982	0.0549	.
F_{ROH}	-0.473 ± 1.576	-0.300	0.7658	
Chron. Age	-0.097 ± 0.066	-1.485	0.146	
Sex	-0.162 ± 0.080	-2.017	0.0510	.
F_{ROH}:Chron. Age	0.0595 ± 0.946	0.063	0.9502	

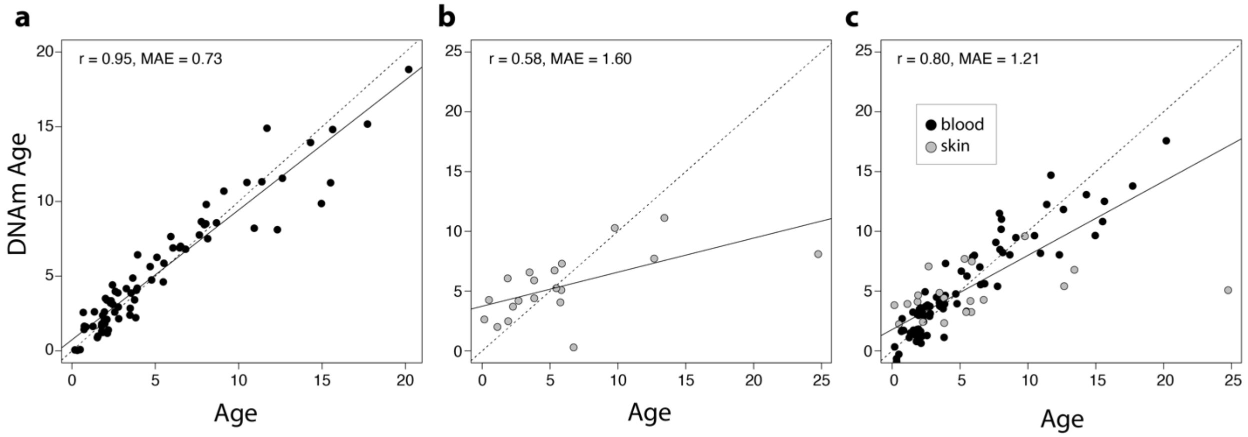

Supplementary Figure 1. Epigenetic clocks using untransformed ages. We developed 3 epigenetic clocks for zebras based on untransformed ages: **a** epigenetic clock for blood samples based on 215 CpG sites, **b** epigenetic clock for skin samples based on 168 CpG sites, and **c** combined tissue clock for both sample types based on 345 CpG sites. Leave-one-sample-out (LOO) estimate of DNA methylation age (y-axis, in units of years) is plotted against chronological age (x-axis). The linear regression of epigenetic age is indicated by a solid line while y=x is depicted by a dashed line. Predictions are based on 76 blood samples and 20 biopsy samples.

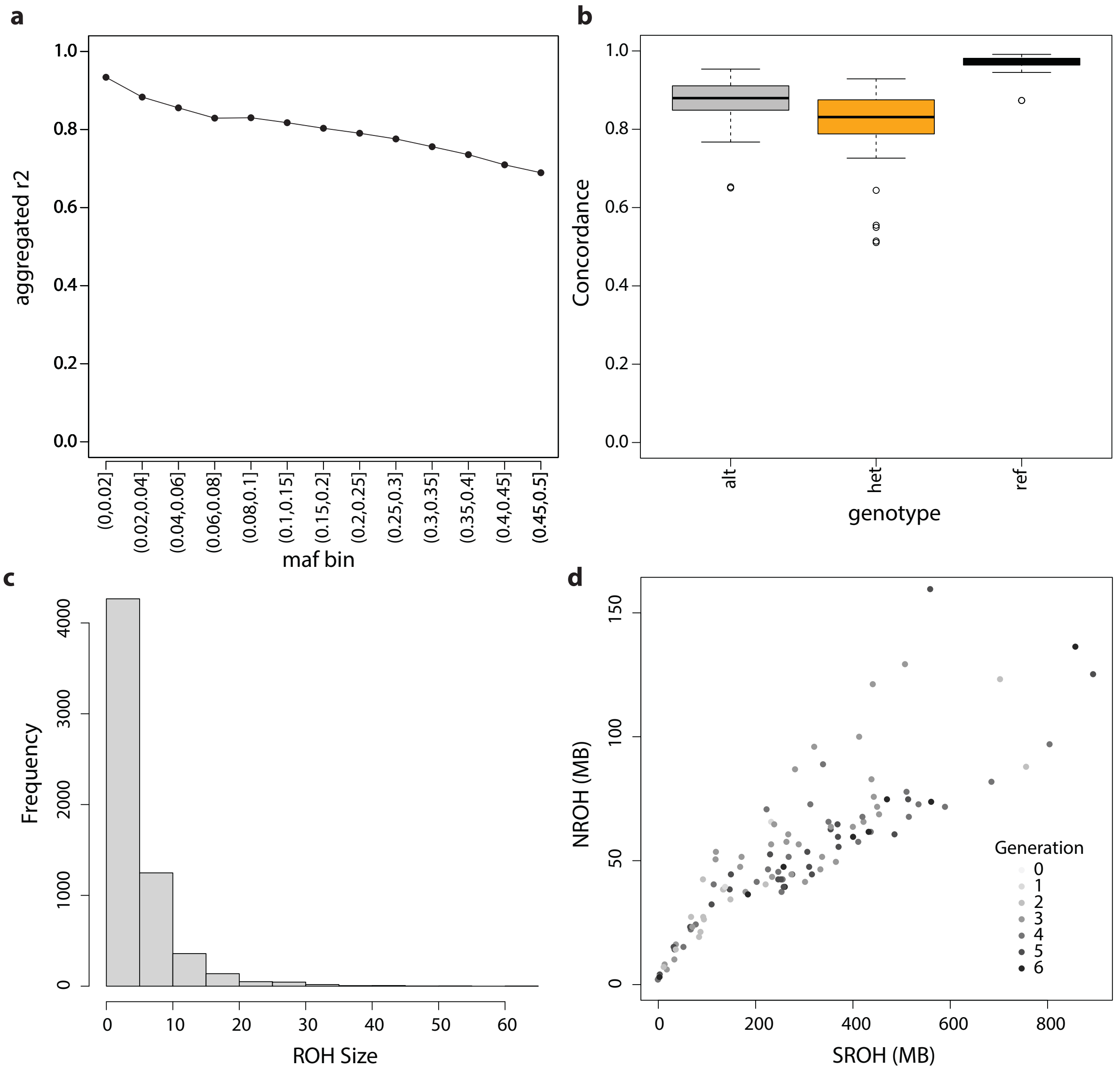

Supplementary Figure 2. Genotype imputation and Distribution of ROH in Quagga Project. **a-b** quality of genotype imputation based on LOO imputations of 35 samples with both RADseq genotypes and genotypes imputed from low coverage sequencing data **a** correlation (r2) between RADseq and imputed genotype dosages is plotted against minor allele frequency, **b** Concordance between imputed and RADseq genotypes. **c** The distribution of ROH size within the samples **d** The number of runs of homozygosity (NROH) in each individual plotted by the sum of the lengths of all homozygous runs found in that individual (SROH). Data are colored by generation in the project, 0 being a founder and 6 being the most recent generation.

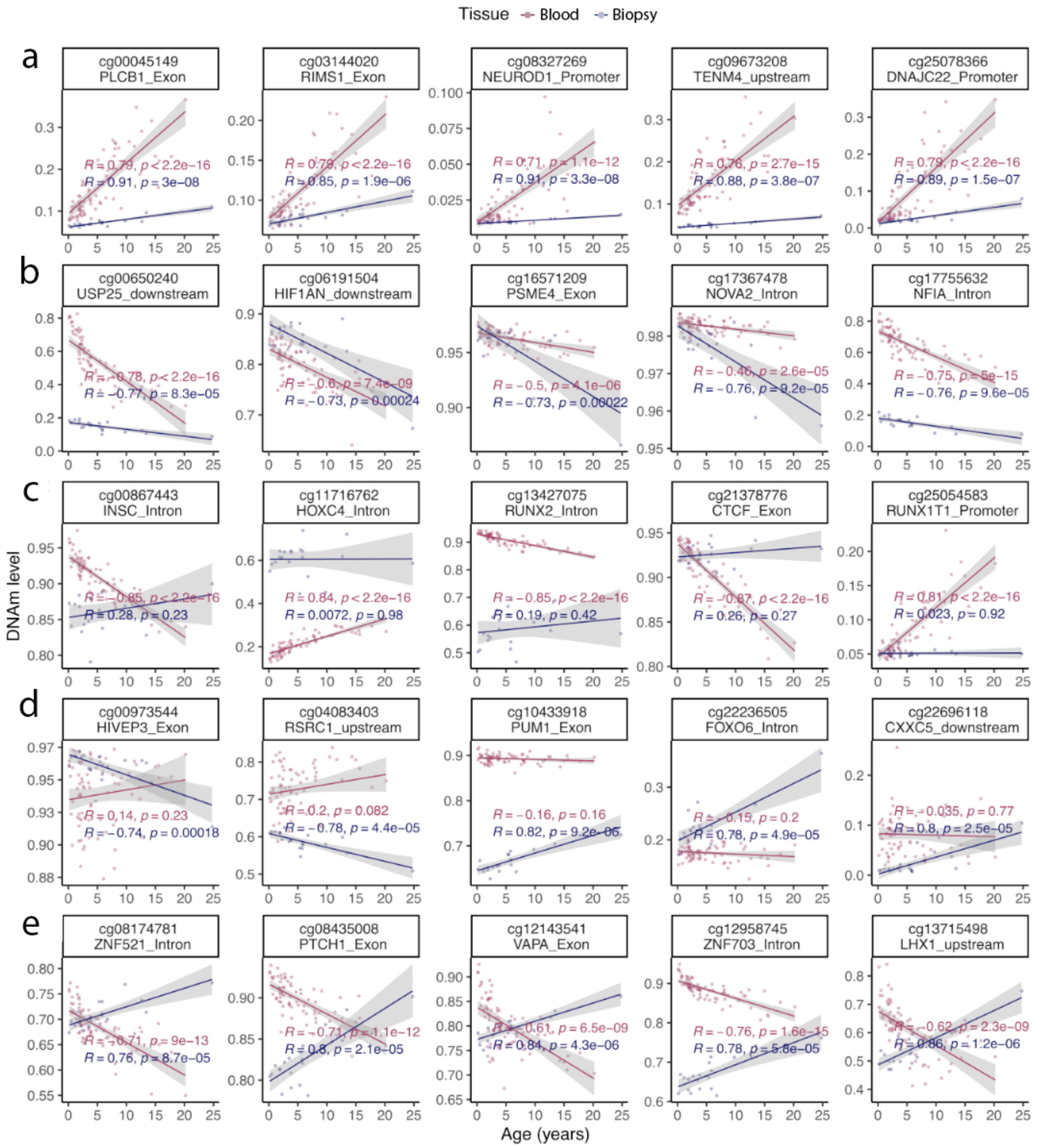

Supplementary Figure 3. Scatter plots of age-related changes in selected CpGs blood and skin of zebras. a CpGs that are hypermethylated with age in both tissues. **b** CpGs that are hypomethylated with age in both tissues. **c** Examples of blood specific changes. **d** Examples of skin specific changes. **e** Selected CpGs with divergent aging pattern between skin and blood of zebras.

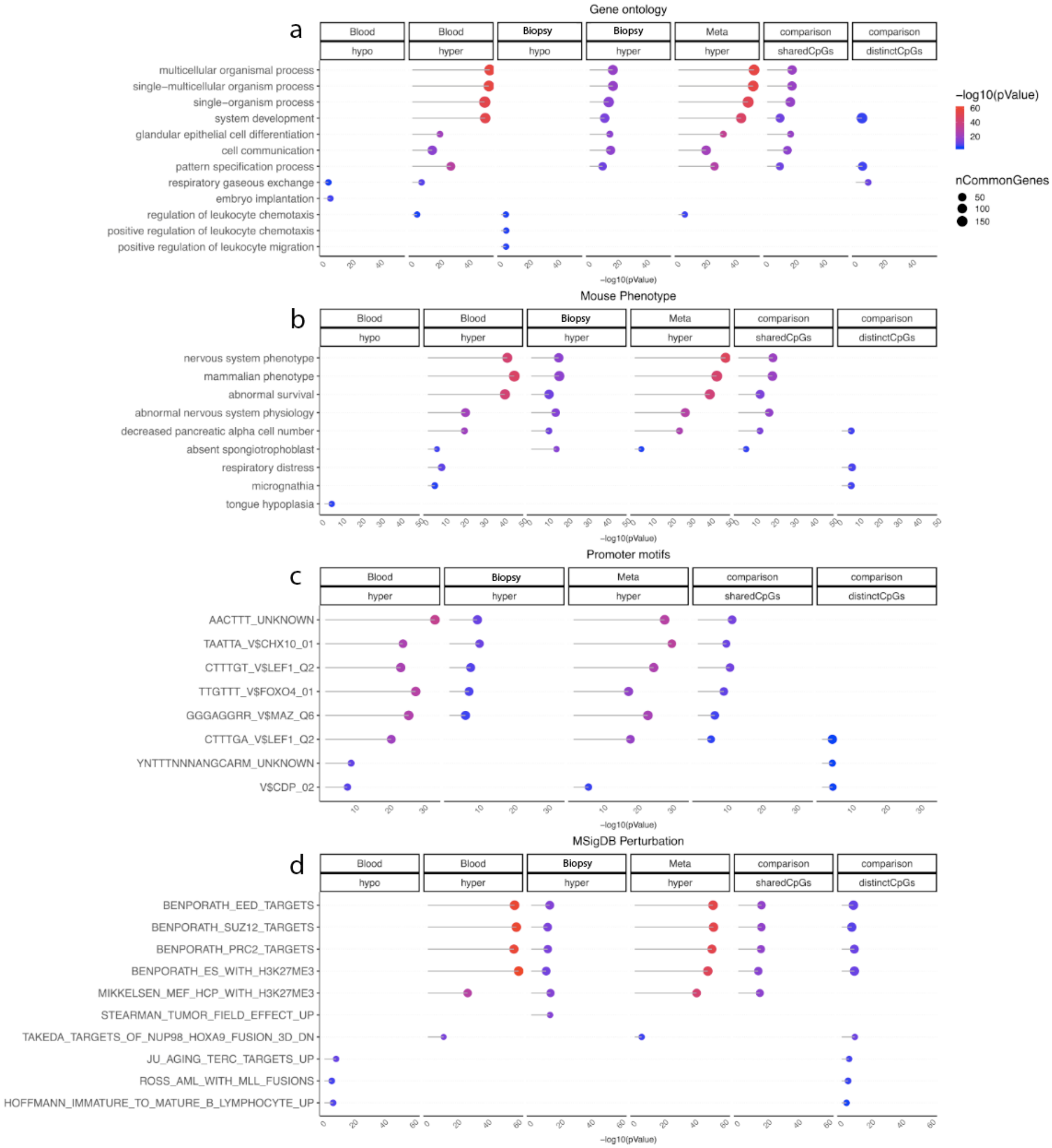

Supplementary Figure 4. Gene set enrichment analysis of DNA methylation aging in zebra. The gene level enrichment was done using GREAT analysis [1] and human Hg19 CpGs that map to the horse genome as background. Datasets: **a** gene ontology, **b** mouse phenotypes, **c** promoter motifs, and **d** MSigDB Perturbation. The results were filtered for significance at $p < 10^{-3}$. Meta refers to the combined blood/biopsy dataset.

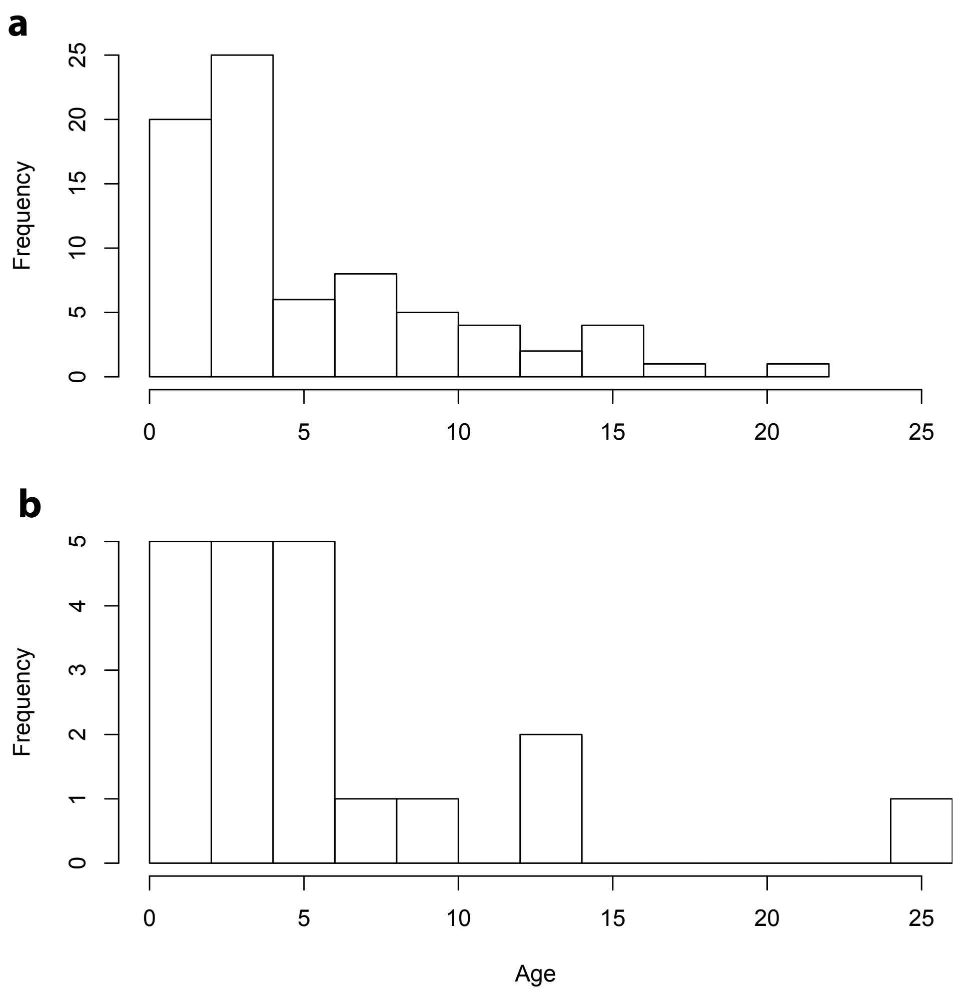

Supplementary Figure 5. Age distribution of the samples. **a** blood samples, **b** skin samples.

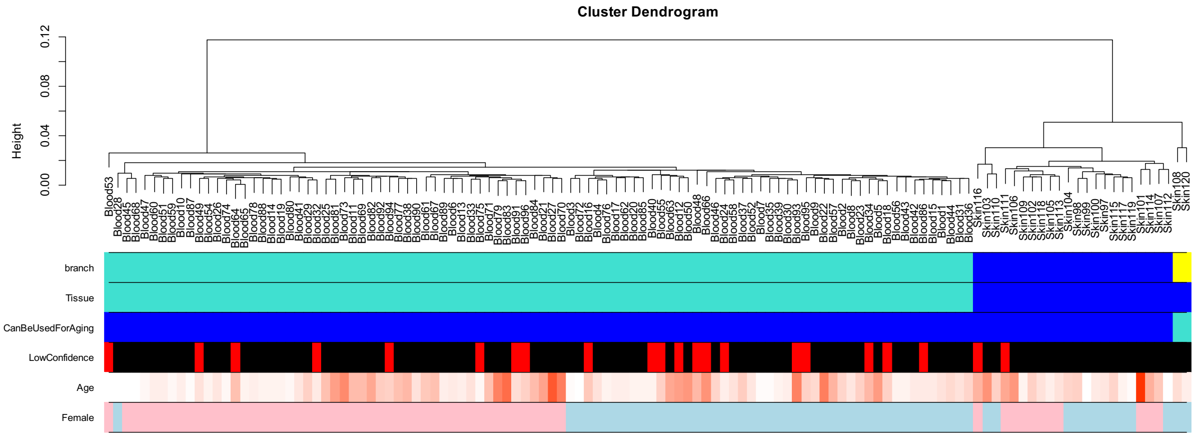

Supplementary Figure 6. **Unsupervised hierarchical clustering of blood and skin samples from zebras.** Average linkage hierarchical clustering based on the inter-array correlation coefficient (Pearson correlation). The relatively low height values (y-axis) indicate high inter array correlations and good quality. However, two skin samples cluster into a distinct yellow cluster (first color band). These putative outliers were removed from the analysis (third color band). Contrasting the first color band (based on cluster branches) with the second color band shows that the arrays cluster by tissue type (blue=skin). The fourth color band indicates wild animals whose ages were largely unknown (low confidence in the provided age estimate). These samples were omitted from the training set. The last color band indicates that the blood samples are grouped by sex.

Supplementary References

1 McLean, C. Y. *et al.* GREAT improves functional interpretation of cis-regulatory regions. *Nat Biotechnol* **28**, doi:10.1038/nbt.1630 (2010).
