# Supplementary material for: Epigenetic models developed for plains zebras predict age in domestic horses and endangered equids
Source: Commun Biol. 2021 Dec 17;4:1412. doi: 10.1038/s42003-021-02935-z (PMC8683477; doi:10.1038/s42003-021-02935-z)
Supplement: Supplementary file 3 — Description of Additional Supplementary Files [file 42003_2021_2935_MOESM3_ESM.pdf]

Description of Additional Supplementary Files

File name: Supplementary Data 1
Description: Pearson coefficients of zebra age on methylation of zebra tissues at horse CpG sites

File name: Supplementary Data 2
Description: CpGs selected by the epigenetic clock and pacemaker models for plains zebras
