# Supplementary material for: Epigenetic models developed for plains zebras predict age in domestic horses and endangered equids
Source: Commun Biol. 2021 Dec 17;4:1412. doi: 10.1038/s42003-021-02935-z (PMC8683477; doi:10.1038/s42003-021-02935-z)
Supplement: Supplementary file 6 — Reporting Summary [file 42003_2021_2935_MOESM6_ESM.pdf]

nature research

Corresponding author(s): *Double-blind peer review submissions: write DBPR and your manuscript number here instead of author names.*

Last updated by author(s): *YYYY-MM-DD*

Reporting Summary

Nature Research wishes to improve the reproducibility of the work that we publish. This form provides structure for consistency and transparency in reporting. For further information on Nature Research policies, see our Editorial Policies and the Editorial Policy Checklist.

Statistics

For all statistical analyses, confirm that the following items are present in the figure legend, table legend, main text, or Methods section.

n/a	Confirmed	
☐	☒	The exact sample size (*n*) for each experimental group/condition, given as a discrete number and unit of measurement
☐	☒	A statement on whether measurements were taken from distinct samples or whether the same sample was measured repeatedly
☐	☒	The statistical test(s) used AND whether they are one- or two-sided<br>*Only common tests should be described solely by name; describe more complex techniques in the Methods section.*
☐	☒	A description of all covariates tested
☐	☒	A description of any assumptions or corrections, such as tests of normality and adjustment for multiple comparisons
☐	☒	A full description of the statistical parameters including central tendency (e.g. means) or other basic estimates (e.g. regression coefficient) AND variation (e.g. standard deviation) or associated estimates of uncertainty (e.g. confidence intervals)
☐	☒	For null hypothesis testing, the test statistic (e.g. *F*, *t*, *r*) with confidence intervals, effect sizes, degrees of freedom and *P* value noted<br>*Give P values as exact values whenever suitable.*
☒	☐	For Bayesian analysis, information on the choice of priors and Markov chain Monte Carlo settings
☒	☐	For hierarchical and complex designs, identification of the appropriate level for tests and full reporting of outcomes
☐	☒	Estimates of effect sizes (e.g. Cohen's *d*, Pearson's *r*), indicating how they were calculated

Our web collection on statistics for biologists contains articles on many of the points above.

Software and code

Policy information about availability of computer code

Data collection	R_4.0.2: Programming language for statistical computing<br>R_sesame_1.3.0: Normalize Illumina Infinium DNA methylation array dat
Data analysis	Rv.4.1.0: Programming language for statistical computing<br>Python 3.7.4: Programming language<br>EpigeneticPacemaker 0.0.350: A fast conditional expectation maximization algorithm for modeling epigenetic state<br>glmnet v.4.0-2: fits generalized linear and similar models via penalized maximum likelihood<br>Freebayes: Variant calling<br>Sentieon: Variant calling<br>BWA: alignment of reads to reference genome<br>GLIMPSE: Genotype imputation<br>VCFTools: Variant filtering<br>PLINK v 1.9: A whole genome analysis toolset we used for estimating F and discovering runs of homozygosity<br>lmtest v.0.9-38 in R: Diagnostic checking in linear regression models<br>sandwich v.3.0-1 in R: Model-robust covariance matrix estimators<br>R_WGCNA_1.69: Weighted correlation network analysis

For manuscripts utilizing custom algorithms or software that are central to the research but not yet described in published literature, software must be made available to editors and reviewers. We strongly encourage code deposition in a community repository (e.g. GitHub). See the Nature Research guidelines for submitting code & software for further information.

Data

Policy information about availability of data

All manuscripts must include a data availability statement. This statement should provide the following information, where applicable:

- Accession codes, unique identifiers, or web links for publicly available datasets
- A list of figures that have associated raw data
- A description of any restrictions on data availability

Methylation data for plains zebras can be downloaded from Gene Expression Omnibus GSE184223. Methylation data for plains zebras can be downloaded from Gene Expression Omnibus GSE184223. RAD sequencing data is available as fastq files on SRA, BioProject ID: PRJNA670933. RADseq and imputed genotypes are available on DRYAD (doi:10.5068/D1W39K).

Field-specific reporting

Please select the one below that is the best fit for your research. If you are not sure, read the appropriate sections before making your selection.

☒ Life sciences ☐ Behavioural & social sciences ☐ Ecological, evolutionary & environmental sciences

For a reference copy of the document with all sections, see nature.com/documents/nr-reporting-summary-flat.pdf

Life sciences study design

All studies must disclose on these points even when the disclosure is negative.

Sample size	Both blood (n=96) and biopsy (skin) (n=24) samples from plains zebras were obtained from a captive population of zebras maintained in a semi-wild state by the Quagga Project (Harley, 2009) in the Western Cape of South Africa. The population was founded in 1989 from 19 individuals (9 from Etosha National Park in Namibia, 10 from the Kwazulu-Natal in South Africa). Skin samples were taken by remote biopsy dart (1 mm wide by 20-25 mm deep plug) and preserved in RNAlater (Qiagen). Blood samples were taken opportunistically during veterinarian visits and preserved in EDTA tubes. Most samples were collected from different individuals, except for two animals that were sampled twice some years apart. All samples were stored at -20 °C. Samples were collected under a protocol approved by the Research Safety and Animal Welfare Administration, University of California Los Angeles: ARC # 2009-090-31, originally approved in 2009. After eliminating samples with low confidence for individual identity and age, we retained 76 blood samples, and 20 skin samples. We retained the founder, however, in an effort to extend the age range represented in the skin clock.<br><br>Additional equid species<br>The data from the 188 horse samples are described in a companion paper (Horvath et al 2021 under review at Communications in Biology) The Grevy's zebra (n=5) and Somali wild ass (n=7) are samples from zoo-based animals that were opportunistically collected and banked during routine health exams. The DNA methylation profiles from these samples have been reported previously in Lu, A. T. et al. Universal DNA methylation age across mammalian tissues. Biorxiv, 2021.2001.2018.426733, doi:10.1101/2021.01.18.426733 (2021).
Data exclusions	None
Replication	All samples were processed as independent biological replicates. We performed a cross-validation scheme for arriving at unbiased (or at least less biased) estimates of the accuracy of the different DNA methylation-based age estimators. One type consisted of leaving out a single sample (LOOCV) from the regression, predicting an age for that sample, and iterating over all samples.<br>Indirect validation of the plains zebra epigenetic clock is afforded by applying it to horses, Grevy's zebras, and Somali ass.
Randomization	This is an observational study, so randomization is not relevant. However, we attempted to randomize samples with respect to age, sex, and tissue source when filling 96-well plates for methylation profiling.
Blinding	Blinding was not relevant to our study, because this is an observational study and all available data were used.

Reporting for specific materials, systems and methods

We require information from authors about some types of materials, experimental systems and methods used in many studies. Here, indicate whether each material, system or method listed is relevant to your study. If you are not sure if a list item applies to your research, read the appropriate section before selecting a response.

Materials & experimental systems

n/a	Involved in the study
☒	☐ Antibodies
☒	☐ Eukaryotic cell lines
☒	☐ Palaeontology and archaeology
☐	☒ Animals and other organisms
☒	☐ Human research participants
☒	☐ Clinical data
☒	☐ Dual use research of concern

Methods

n/a	Involved in the study
☒	☐ ChIP-seq
☒	☐ Flow cytometry
☒	☐ MRI-based neuroimaging

Animals and other organisms

Policy information about studies involving animals; ARRIVE guidelines recommended for reporting animal research

Laboratory animals

Horses. The details of the horse samples used to validate the plains zebra clock are reported in a companion paper (Horvath, 2021 in review at Communications in Biology). In brief, most of the samples were from the Thoroughbred (TB) (n=79) and American Quarter Horse breeds (QH, n=62). For the following breeds, we had between one and six blood samples: Andalusian, Appaloosa, Arabian, Dutch Warmblood, Hanoverian, Holsteiner, Irish Sport Horse, Lipizzaner, Lusitano, mixed breed, Oldenburg, Paint or Paint cross, Percheron, Shire, Standardbred, Warmblood and Welsh Pony. The n=49 liver samples originated from necropsy collections of horses across 19 different breeds, with most of the liver samples from QHs (n=20). All collection protocols were approved by the UC Davis Institutional Animal Care and Use Committee (Protocols #20751 and 21455, respectively).

Wild animals

Both blood (n=96) and biopsy (skin) (n=24) samples from plains zebras were obtained from a captive population of zebras maintained in a semi-wild state by the Quagga Project (Harley, 2009)n the Western Cape of South Africa. The population was founded in 1989 from 19 individuals (9 from Etosha National Park in Namibia, 10 from the Kwazulu-Natal in South Africa). Skin samples were taken by remote biopsy dart (1 mm wide by 20-25 mm deep plug) and preserved in RNAlater (Qiagen). Blood samples were taken opportunistically during veterinarian visits and preserved in EDTA tubes. Most samples were collected from different individuals, except for two animals that were sampled twice some years apart. All samples were stored at -20 °C. Samples were collected under a protocol approved by the Research Safety and Animal Welfare Administration, University of California Los Angeles: ARC # 2009-090-31, originally approved in 2009. After eliminating samples with low confidence for individual identity and age, we retained 76 blood samples, and 20 skin samples. We retained the founder, however, in an effort to extend the age range represented in the skin clock. The mean age of blood was 5.2 years ranging from 0.16 years to 20 years. The mean age of skin was 5.9 years ranging from 0.16 years to 25 years.

The Grevy's zebra (n=5) and Somali wild ass (n=7), are samples from zoo-based animals that were opportunistically collected and banked during routine health exams and the DNA methylation profiles from these samples have been reported previously in Lu, A. T. et al. Universal DNA methylation age across mammalian tissues. Biorxiv, 2021.2001.2018.426733, doi:10.1101/2021.01.18.426733 (2021).

Field-collected samples

same as wild animals

Ethics oversight

Horse: UC Davis Institutional Animal Care and Use Committee (Protocols #20751 and 21455, respectively).
Other equids. Research Safety and Animal Welfare Administration, University of California Los Angeles: ARC # 2009-090-31

Note that full information on the approval of the study protocol must also be provided in the manuscript.
